# Using Dual-Orthogonal Fluoroscopy and CT to Assess the Relationship Between Knee Morphology and Patellar Kinematics in Patients With Patellofemoral Pain

**DOI:** 10.7759/cureus.44139

**Published:** 2023-08-25

**Authors:** Joanna Yuen, Fateme Esfandiarpour, Constance M Lebrun, Suki Dhillon

**Affiliations:** 1 Department of Radiology, University of British Columbia, Vancouver, CAN; 2 Department of Family Medicine, University of Alberta, Edmonton, CAN; 3 Department of Sport and Exercise Medicine, MacEwan University Health Center, Edmonton, CAN; 4 Department of Radiology, University of Alberta, Edmonton, CAN

**Keywords:** knee morphology, patellofemoral pain, fluoroscopy, ct, knee mal-tracking

## Abstract

Introduction: Patellofemoral pain (PFP) is one of the most common knee overuse injuries, with studies suggesting PFP as a precursor for early knee osteoarthritis. The etiology of PFP is multi-factorial; however, patellar mal-tracking has been regarded as a primary mechanism. Details of this multi-factorial mechanism have been unclear because of the limitations in evaluating in-vivo, three-dimensional (3D) patellofemoral joint movement during dynamic activities accurately. Alternatively, studies have demonstrated the high accuracy and repeatability of dual fluoroscopy and CT/MRI for measuring knee joint motion.

Objective: This study uses dual fluoroscopy and CT to investigate the associations between joint morphology and patellar kinematics in healthy controls and subjects with PFP.

Methods: Eight PFP females (29.7±10.6 years) and 10 healthy females (25.0±7.7 years) were recruited and screened by a sports medicine physician. CT imaging was performed on participants in a supine with the knee extended, and ankle and hip in neutral alignment. Dual-orthogonal fluoroscopy measured patellar movement while participants performed a lunge task. A calibration algorithm was used to register the 3D CT model to 2D fluoroscopy image to calculate the relative position and angles of the patella based on the clinical definition of patellar motion. Measures of patellar and trochlear morphology were generated and correlated to kinematic data.

Results and conclusion: There was a significant difference in the patellar-to-trochlear width ratio; however, no other significant differences in CT morphology measurements were present between groups. For PFP patients in the weight-bearing extended position, there was a moderate positive correlation between the patellar-to-trochlear width ratio and medial-lateral patellar shift (τ = 0.643, p = 0.026). Healthy controls in this position demonstrated a moderate positive correlation between the lateral-trochlear inclination angle and medial-lateral patellar shift (τ = 0.600, p = 0.016) and moderate negative correlation between medial trochlear inclination angle and medial-lateral patellar shift (τ = -0.511, p = 0.040). The findings suggest that, for this cohort, there is correlation between morphology and patellar kinematics. Passive and active stabilizers likely have a role in mal-tracking.

## Introduction

Patellofemoral pain (PFP) is one of the most common knee overuse injuries amongst physically active individuals, with a higher prevalence in females [1]. Characterized as peri-patellar or retro-patellar pain, PFP is exacerbated by movements including deep knee flexion, repetitive flexion, extension, or prolonged sitting. As a result, PFP patients generally reduce or restrict common activities, such as running, jumping, and ascending and descending stairs [2]. As PFP often recurs or persists for many years, studies have suggested PFP as a precursor for early osteoarthritis [2,3]. Thus, developing an understanding of the causes of PFP can have implications for the prevention and treatment of this condition.

The etiology of PFP is multi-factorial, involving anatomical, biomechanical, psychological, and behavioral factors. Patellar mal-tracking has been regarded as a primary mechanism for PFP [4-7]. The abnormal mechanics of patellar mal-tracking result in a pathological change in stress distribution and joint pain [5]. Active and passive patellar stabilizers such as joint morphology, muscle force, and tendon activity may influence these mechanics of patellar mal-tracking [8-11]. However, details of this multi-factorial mechanism have been unclear because of limitations of evaluating in-vivo, three-dimensional (3D) patellofemoral joint movement during dynamic activities.

Video motion analysis is one of the most common methods of studying human joint motion. The movement of surface markers corresponds to the kinematics of the underlying bony segments. However, this association is affected by skin deformation, gravity, and inertial effects, which in turn, causes soft tissue artifacts [12]. In addition to limiting accuracy, soft tissue artifacts cause poor resolution in the coronal and axial planes [13]. Imaging techniques to study the knee joint is a solution for improving accuracy in measurement. These techniques have been used widely for studying knee joint motion in the last decade. They have evolved from static two-dimensional (2D) imaging to more dynamic imaging of joint motion, such as cine-phase magnetic resonance imaging (MRI) and real-time MRI. Although cine-phase MRI can provide 3D analysis, it is limited to non-weight-bearing activities in a supine or prone position over a small range of motion [14,15]. In comparison, real-time MRI enables measurements over a greater range of motion and during functional movements. However, it is limited to 2D analysis during relatively slow movement and has a measurement accuracy of 1.9 mm, preventing it from detecting minor motion [16].

Alternatively, studies have demonstrated high accuracy (0.1 mm translation, 0.1° orientation) and repeatability of dual fluoroscopy and CT/MRI for measuring 6 degrees of freedom (DoF) in knee joint motion [17,18]. Two fluoroscopes are oriented so that the entire knee motion during a weight-bearing task could be captured by both fluoroscopes. A calibration algorithm is then used to register the fluoroscopic images with 3D anatomic knee joint models to determine 6 DoF kinematics. These recent studies have applied this combined fluoroscopic and MR/CT methodology for investigating patellar motion in patients with pathology including PFP, anterior cruciate ligament deficiency, and total knee arthroplasty [19-21]. Notably, Esfandiarpour et al. used this technique to study 6 DoF patellar motion while healthy and PFP participants performed a lunge. They reported the patella being significantly laterally tilted and more superior shifted in knee extension (supine and upright) and during a dynamic lunge in PFP patients [21].

The current study explored the relationship between bony CT morphology in the same cohort of PFP patients and healthy controls. The study investigated correlations between patellar and trochlear morphology and 6 DoF patellofemoral kinematics. Based on prior studies, the present study hypothesized that joint morphology is a factor for patellar mal-tracking [9,22].

## Materials and methods

Data collection and processing

This level II retrospective study is a continuation of a previous study by Esfandiarpour et al. [21]. It received ethics approval from the University Research Ethics Board (approval number: Pro00071298). Eight female PFP patients and 10 female controls were recruited and screened by a sports medicine physician (Table 1). The inclusion criteria for the PFP cohort included having a history of peri-patellar or retro-patellar knee pain with insidious onset aggravated by ascending or descending stairs, squatting, kneeling, jumping, or long-sitting and running. Each knee had a pain severity of at least 30 on the 100-mm Visual Analogue Scale within the last three months. All patients provided written informed consent prior to testing.

**Table 1:** Participant demographics (mean± standard error of the mean) PFP, patellofemoral pain.

Parameter	Age (years)	Weight (kg)	Height (cm)
PFP patients (n=8)	29.7 ± 10.6	63.0 ± 9.1	165.6 ± 7.6
Controls (n=10)	25.0 ± 7.7	61.6 ± 7.4	65.8 ± 4.5

Kinematic measurements

In the General Electric CT scanner, participants were positioned supine with the knee extended and ankle and hip joint in neutral position. The affected or most affected knee of PFP patients or a randomly selected knee of healthy controls were imaged (25 cm field of view, voxel size of 0.488 x 0.477 x 0.625 mm^3^, 0.625 mm spacing between slices, and resolution of 512 x 523 x 256 pixels).

A dual-orthogonal fluoroscopic system (Seimens; 71 kvp, 1.9 mA, average dose rate of 1.163 mRem/min) captured the motion of the knee joint during a lunge from full knee extension to at least 90° flexion. Participants performed a 5-min walking warm-up and two to three practices of the lunge task before one trial of fluoroscopic measurement.

A 3D CT model of each joint was created by a custom MATLAB program (The MathWorks, Inc., Natick, MA). The 3D CT model was then registered to the 2D fluoroscopy images using a calibration algorithm in Fluomotion software (Innomotion, Inc., Shanghai, China) [17,18]. Details about the 3D-to-2D registration algorithm are described by Esfandiarpour et al. and Li et al. [17,21]. For each flexion angle, the location and orientation of each bone were adjusted to match the bone boundaries of the fluoroscopic images. The relative position and angles were calculated to obtain 6 DoF of patellar movement as the joint moved from extension to 75° flexion [21]. Furthermore, 3D patellar orientation and position with respect to the femur were determined in the supine knee extended position on 3D CT models.

Morphology measurements

Trochlear and patellar morphology was assessed by a reader who was blinded to the cohort grouping. Measurements were made on CT images using ImageJ (NIH, Bethesda, MD) and were repeated twice with over 48 hours in between measurements [9]. For each joint, the femoral-axial reference slice was defined as the section with the greatest trans-epicondylar axis and was used to make measurements of femoral trochlear geometry.

**Figure 1:**
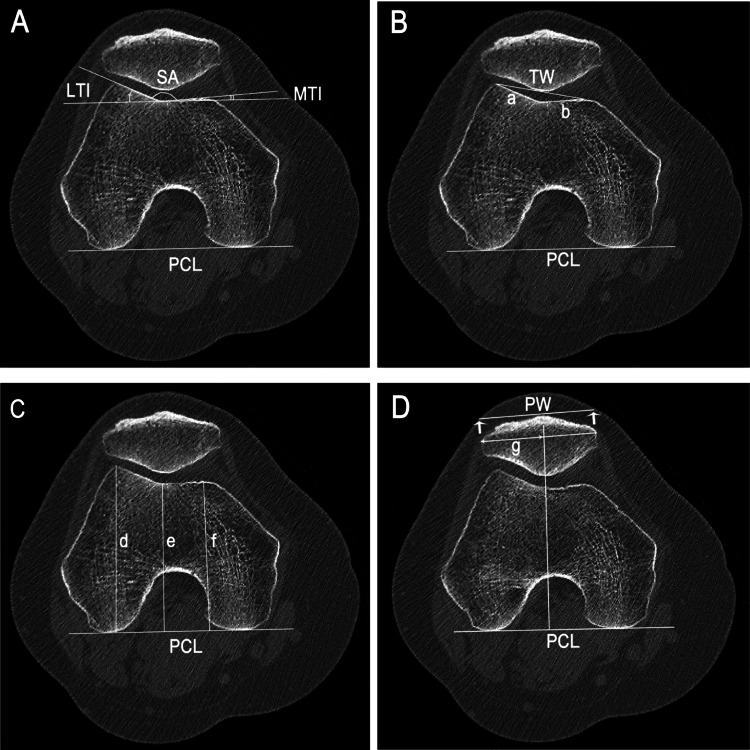
Femoral and patellar morphology measurements. A) Lateral trochlear inclination (LTI) is the angle between the line along the lateral facet to the posterior condylar line (PCL). Medial trochlear inclination (MTI) is the angle between the line along the medial facet and the PCL. (B) Lateral facet (a) and medial facet (b) are regarded as the lengths between the most anterior prominence on the lateral and medial facets, respectively, to the deepest point of the trochlear groove. (C) Trochlear depth is defined as (d+f)/2-e, where f, d, and e are the “heights” of the medial condyle, lateral condyle, and sulcus groove, respectively. The respective “heights” are measured as the perpendicular distances from the most anterior medial and lateral prominence and deepest point of the trochlear groove to the PCL. Condylar asymmetry is the ratio between the medial condylar height (f) and lateral condylar height (d). (D) Patellar width (PW) is the length between the most lateral and medial aspects of the patella. Lateral PW (g) is defined as the length between the most lateral aspect of the patellar to the line perpendicular to the deepest point of the trochlear groove. SA - Sulcus angle

Measures of patellar geometry were conducted on the patellar-axial reference slice, the section with the greatest patellar width (PW). In both sections, the posterior condylar line (PCL) was drawn to connect the medial and lateral posterior femoral condyles. Lateral trochlear inclination (LTI) was defined as the angle between the lateral facet to the PCL (Figure 1A). The medial trochlear inclination (MTI) was the angle between the medial facet and the PCL (Figure 1A). Sulcus angle (SA) was measured as the angle between the medial and lateral facets. Lateral facet and medial facet lengths were measured from the most anterior prominence of the lateral and medial facets respectively to the deepest point of the trochlear groove (Figure 1B). The ratio of these two lengths was used to calculate facet asymmetry. Trochlear width (TW) was the length between the most anterior lateral prominence to the most anterior medial prominence (Figure 1B). Trochlear depth ((d+f)/2-e) was calculated by the heights from the medial condyle (f), lateral condyle (d), and sulcus groove (e) to the PCL respectively (Figure 1C). Condylar asymmetry was calculated as ratio between the medial condylar height and lateral condylar height. PW was the length between the most lateral and medial aspects of the patella (Figure 1D). Lateral PW was measured from the most lateral aspect of the patellar to the line perpendicular to the deepest point of the trochlear groove. PW-to-TW ratio was calculated as the ratio of the PW to TW.

Statistical analysis

Statistical analysis was performed using SPSS statistical software (version 25; IBM Corp., Armonk, NY). Significance levels were set at p < 0.05. The intraclass correlation coefficients were calculated to assess intra-rater reliability. Independent t-tests (two-tailed, unequal variances) were used to compare morphological parameters between healthy controls and PFP patients. All measures were scaled by the ratio of the healthy control’s average trans-epicondylar width (75.8 mm) to each participant’s trans-epicondylar width [9]. Correlations (> 0.5 defined as predictive) were measured with the values of each kinematic variable (Kendall’s τ, two-tailed) during a lunge, from the starting knee extended position to 75° flexion. Correlations were also measured at the supine, non weight-bearing knee extended position.

## Results

Morphology measurements

With the exception of the PW-to-TW ratio, all morphology measurements had good intra-observer reliability, with intraclass coefficient coefficients (ICC) ranging from 0.89 to 0.98 (Table 2). The PW-to-TW ratio was the least reliable, albeit still acceptable, with an ICC and Cronbach’s alpha of 0.790 and 0.780, respectively.

**Table 2:** Intra-rater reliability for CT morphology with greater than 48 hours between measurements. LTI, lateral trochlear inclination; MTI, medial trochlear inclination; SA, sulcus angle; TD, trochlear depth; PTR, patellar-to-trochlear width ratio; CA, condylar asymmetry; FA, facet asymmetry; LPW, lateral patellar width.

Parameter	LTI	MTI	SA	TD	PTR	CA	FA	LPW
Intraclass correlation coefficient	0.976	0.932	0.919	0.983	0.790	0.987	0.893	0.942
Cronbach’s Alpha	0.983	0.937	0.917	0.982	0.780	0.974	0.896	0.939

There was a significant difference between groups in the PW-to-TW ratio (p = 0.043). No other significant differences in CT morphology measurements were present between groups (Table 3).

**Table 3:** CT morphology measurements (mean± standard error of the mean). PFP, patellofemoral pain; LTI, lateral trochlear inclination; MTI, medial trochlear inclination; SA, sulcus angle; TD, trochlear depth; PTR, patellar-to-trochlear width ratio; CA, condylar asymmetry; FA, facet asymmetry; LPW, lateral patellar width.

Feature	Knees with PFP	Healthy knees	P-value
LTI (°)	19.85 ± 1.52	18.26 ± 2.00	NS
MTI (°)	20.95 ± 2.42	20.63 ± 8.83	NS
SA (°)	142.43 ± 14.48	145.11 ± 8.97	NS
TD (mm)	5.50 ± 1.24	4.39 ± 1.07	NS
PTR (ratio)	1.25 ± 0.17	1.45 ± 0.21	0.043
CA (ratio)	0.94 ± 0.06	0.94 ± 0.06	NS
FA (ratio)	2.40 ± 1.11	2.41 ± 0.91	NS
LPW (mm)	28.78± 5.13	27.44 ± 5.37	NS

Patellar kinematics and morphology

In the starting knee-extended position (Table 4), PFP patients demonstrated positive correlations between the PW-to-TW ratio and medial-lateral patellar shift (τ = 0.643, p = 0.026) and lateral PW and medial-lateral patellar shift (τ = 0.714, p = 0.013). In healthy controls at this extended position (Table 5), there was a moderate positive correlation between the lateral trochlear inclination angle and medial-lateral patellar shift (τ = 0.600, p = 0.016). There was also a moderate negative correlation between medial trochlear inclination angle and medial-lateral patellar shift (τ = -0.511, p = 0.040). For PFP patients doing a lunge, a moderate positive correlation between the PW-to-TW ratio and patellar tilt was apparent at 60° flexion (τ = 0.571, p = 0.048).

**Table 4:** Correlation between morphology and PF joint orientation and position for PFP patients in a standing position. τ, Kendall’s tau; LTI, lateral trochlear inclination; MTI, medial trochlear inclination; SA, sulcus angle; TD, trochlear depth; PTR, patellar-to-trochlear width ratio; CA, condylar asymmetry; FA, facet asymmetry; LPW, lateral patellar width; ML, medial-lateral patellar shift; AP, anterior-posterior patellar shift; SI, superior-inferior patellar shift.

		Flex	Rot	Tilt	ML	AP	SI
LTI	τ	0.286	0	0	-0.286	-0.071	-0.071
	P-value	0.322	1	1	0.322	0.805	0.805
MTI	τ	-0.143	-0.286	0	0.286	-0.071	0.214
	P-value	0.621	0.322	1	0.322	0.805	0.458
SA	τ	-0.143	0.429	-0.143	-0.143	-0.071	-0.214
	P-value	0.621	0.138	0.621	0.621	0.805	0.458
TD	τ	0.214	-0.214	-0.071	-0.071	-0.143	0
	P-value	0.458	0.458	0.805	0.805	0.621	1
PTR	τ	-0.357	0.357	0.214	0.643	0.143	0.143
	P-value	0.216	0.216	0.458	0.026*	0.621	0.621
CA	τ	-0.071	-0.5	0.071	0.357	0	0.143
	P-value	0.805	0.083	0.805	0.216	1	0.621
FA	τ	-0.643	0.357	0.071	0.357	0	0.286
	P-value	0.026*	0.216	0.805	0.216	1	0.322
LPW	τ	-0.429	-0.143	0.286	0.714	0.214	0.214
	P-value	0.138	0.621	0.322	0.013*	0.458	0.458

**Table 5:** Correlation between morphology and PF joint orientation and position for healthy controls in a standing position. τ, Kendall’s tau; LTI, lateral trochlear inclination; MTI, medial trochlear inclination; SA, sulcus angle; TD, trochlear depth; PTR, patellar-to-trochlear width ratio; CA, condylar asymmetry; FA, facet asymmetry; LPW, lateral patellar width; ML, medial-lateral patellar shift; AP, anterior-posterior patellar shift; SI, superior-inferior patellar shift.

		Flex	Rot	Tilt	ML	AP	SI
LTI	τ	-0.200	0.289	-0.289	0.600	-0.156	0.378
	P-value	0.421	0.245	0.245	0.016*	0.531	0.128
MTI	τ	0.467	-0.289	0.200	-0.511	0.067	-0.200
	P-value	0.060	0.245	0.421	0.040*	0.788	0.421
SA	τ	-0.556	0.467	-0.111	0.422	-0.067	0.200
	P-value	0.025*	0.060	0.655	0.089	0.788	0.421
TD	τ	0.067	0.200	-0.111	0.333	-0.156	0.467
	P-value	0.788	0.421	0.655	0.180	0.531	0.060
PTR	τ	0.156	-0.422	-0.378	-0.200	-0.333	-0.511
	P-value	0.531	0.089	0.128	0.421	0.180	0.040*
CA	τ	0.422	-0.244	0.067	-0.467	0.022	-0.422
	P-value	0.089	0.325	0.788	0.060	0.929	0.089
FA	τ	-0.067	0.156	0.378	-0.156	0.156	0.333
	P-value	0.788	0.531	0.128	0.531	0.531	0.180
LPW	τ	0.200	-0.022	0.467	0.733	0.244	-0.200
	P-value	0.421	0.929	0.060	0.003*	0.325	0.421

For PFP patients in the non-weight bearing extended position, there were no correlations between morphology and patellar alignment. However, the healthy group demonstrated a moderate positive correlation between lateral PW and medial-lateral patellar shift (τ = 0.600, p = 0.016).

## Discussion

As per the study hypothesis, the findings of this study suggest a correlation between morphology and patellar kinematics. There was a significant difference in the PW-to-TW ratio between healthy controls and PFP patients. Interestingly, in the PFP group, the results also showed positive correlations between the PW-to-TW ratio and medial-lateral patellar shift at full knee extension and between the PW-to-TW ratio and patellar tilt at 60° flexion.

Current proposed factors for patellar mal-tracking include altered knee morphology, hip joint alignment, muscle force, and soft tissue restraints [22]. Previously, Esfandiarpour et al. had reported an abnormal lateral patellar tilt and superior translation in the weight-bearing and non-weight-bearing extended positions and during the lunge in individuals with PFP [21]. For this same cohort, the current study demonstrated that there was a significant difference between the PW-to-TW ratio between groups. No other measures in CT morphology were significantly different.

Prior studies based on static 2D imaging have reported differences in patellar height, SA, and sulcus groove length [9,22,23-25]. In most studies, subjects with increased trochlear angle were more likely to have superolateral Hoffa fat pad edema [26]. On the other hand, Drew et al. reported no statistically significant differences in patellofemoral imaging features among groups with and without PFP [27]. Similarly, Harbaugh et al. found no differences in trochlear dysplasia (trochlear groove width, facet asymmetry, condylar asymmetry), PW, and the trochlear to patellar ratio between PFP and health cohorts [9]. After subdividing PFP patients into lateral and non-lateral mal-trackers, the authors reported that many of the morphology parameters lie on opposite sides of the healthy mean [9]. Thus, these subgroups may be masking the morphological differences between cohorts. However, due to the current study size, it was not possible to further subdivide mal-trackers for subsequent further analysis and verification. On the other hand, it is possible that active patellar stabilizers, such as muscle and tendon function, contribute to patellar mal-tracking.

Several studies have investigated kinematics and morphology in patients with PFP and have provided support that there are differences in all three planes of motion [22,24]. However, these differences have not been consistent. For instance, while some studies have reported the inclination of the lateral anterior femoral condyle and SA being correlated with mediolateral patellar tracking, others found minimal correlations between those parameters [9,11,22,28]. One possibility for the inconsistency may be due to past studies previously using 2D static measures of patellofemoral alignment to predict three-dimensional PF kinematics. These 2D measures are not accurate in predicting 3D kinematics and have been reported to be highly variable, with r^2^ values ranging from 16% to 77% [29]. The present study used a highly accurate fluoroscopic method with excellent repeatability to measure 6 DoF patellar motion. PFP patients demonstrated a moderate positive correlation between the PW-to-TW ratio and medial-lateral patellar shift (τ = 0.643, p = 0.026) at full knee extension during a lunge. This cohort also had a positive correlation between the PW-to-TW ratio and patellar tilt at 60° of flexion (τ = 0.571, p = 0.048). For the PFP cohort in a supine non-weight-bearing, knee-extended position, in which muscular forces likely do not play a major role in patellar orientation, there were no significant correlations between kinematic and morphology parameters. This suggests that investigating patellar orientation in the weight-bearing or weight-loaded condition may provide more relevant information about patellar orientation, compared to the non-weight-bearing supine lying position that is currently being performed in clinical practice.

This study’s primary limitation is its small sample size, in which potential measurement outliers may have a greater effect on statistical analysis. A prior pilot study, using a sample-size power analysis of β = 0.2 and α = .05 on the mean differences for patellar tilt, determined that a maximum of seven participants per group was needed to power this study adequately [21]. Furthermore, there was good intra-observer reliability in the present study, in accordance with previous studies [30].

## Conclusions

In conclusion, the study's findings demonstrate that for this patient cohort, there is a correlation between morphology and patellar kinematics. Passive and active stabilizers likely have a role in mal-tracking. Future studies should examine the role of a muscle action sequence and the association of proximal and distal kinematic factors (hip, ankle) in patellar kinematics. This is important in correctly developing a thorough understanding of the pathophysiology and eventual treatment of PFP.
